# Photosynthetic Carbon Partitioning and Metabolic Regulation in Response to Very-Low and High CO_2_ in *Microchloropsis gaditana* NIES 2587

**DOI:** 10.3389/fpls.2020.00981

**Published:** 2020-07-03

**Authors:** Mukul Suresh Kareya, Iqra Mariam, Kashif Mohd Shaikh, Asha Arumugam Nesamma, Pannaga Pavan Jutur

**Affiliations:** Omics of Algae Group, Industrial Biotechnology, International Centre for Genetic Engineering and Biotechnology, New Delhi, India

**Keywords:** *Microchloropsis*, carbon dioxide, oleaginous microalga, biomass, photosynthetic cell factories

## Abstract

Photosynthetic organisms fix inorganic carbon through carbon capture machinery (CCM) that regulates the assimilation and accumulation of carbon around ribulose-1,5-bisphosphate carboxylase/oxygenase (Rubisco). However, few constraints that govern the central carbon metabolism are regulated by the carbon capture and partitioning machinery. In order to divert the cellular metabolism toward lipids and/or biorenewables it is important to investigate and understand the molecular mechanisms of the CO_2_-driven carbon partitioning. In this context, strategies for enhancement of CO_2_ fixation which will increase the overall biomass and lipid yields, can provide clues on understanding the carbon assimilation pathway, and may lead to new targets for genetic engineering in microalgae. In the present study, we have focused on the physiological and metabolomic response occurring within marine oleaginous microalgae *Microchloropsis gaditana* NIES 2587, under the influence of very-low CO_2_ (VLC; 300 ppm, or 0.03%) and high CO_2_ (HC; 30,000 ppm, or 3% v/v). Our results demonstrate that HC supplementation in *M. gaditana* channelizes the carbon flux toward the production of long chain polyunsaturated fatty acids (LC-PUFAs) and also increases the overall biomass productivities (up to 2.0 fold). Also, the qualitative metabolomics has identified nearly 31 essential metabolites, among which there is a significant fold change observed in accumulation of sugars and alcohols such as galactose and phytol in VLC as compared to HC. In conclusion, our focus is to understand the entire carbon partitioning and metabolic regulation within these photosynthetic cell factories, which will be further evaluated through multiomics approach for enhanced productivities of biomass, biofuels, and bioproducts (B3).

## Introduction

Environmental pollution by the greenhouse emissions ultimately led to global warming and accumulation of 440 ppm of CO_2_ which is one of the significant gas released into the atmosphere contributing for adverse environmental issues (Ng et al., 2018). The use of fossil fuels is primarily in three economic sectors, namely: energy, transportation, and industry leading to CO_2_ emissions. Increase in atmospheric CO_2_ may be caused due to following reasons such as deforestation (9%), burning of fossil fuels (87%), and remaining (4%) presumably by others like industrial manufacturing (Le Quere et al., 2013; Mistry et al., 2019).

Microalgae are unicellular photosynthetic microbes capable of converting atmospheric CO_2_ into lipids (Ho et al., 2010; Mata et al., 2010; Chen et al., 2011) and other high-valuable renewables (Wang et al., 2010). Most of the eukaryotic algae comprises of pyrenoids, which are proteinaceous sub-cellular compartmentalized structures capable of fixing nearly 30–40% of atmospheric CO_2_ due to the presence of Rubisco enzyme (Freeman Rosenzweig et al., 2017). Pyrenoids are considered as the hubs for carbon assimilation in algae and their structural characteristics may vary depending upon the species (Raven et al., 2008). Capturing of the atmospheric CO_2_ and converting them into reduced form without contributing to global warming by maintaining the balance in the environment is referred as carbon sequestration (Lal, 2008; Zeng, 2008). These photosynthetic cell factories are capable of sequestering atmosphere CO_2_ for the production of biofuel precursors (Rittmann, 2008; Mistry et al., 2019). Henceforth, the potential of industrially relevant oleaginous microalgae to minimize the excess CO_2_ present in the atmosphere can be employed *via* carbon concentrating mechanism (CCM) (Long et al., 2016).

Broadly three types of CCMs are reported in plants and algae, i.e., C3, C4, and crassulacean acid metabolism (CAM) (Hopkinson et al., 2016; Heyduk et al., 2019). For example, in model microalgae *Chlamydomonas reinhardtii* and other diatoms, presence of below air-level CO_2_ especially activates CCMs which are quite sensitive (Reinfelder, 2011; Meyer and Griffiths, 2013; Wei et al., 2019). Even though each species may have distinct CCMs due to their diversity, till date it is not clearly defined whether these diverse CCMs are universally occurring in all microalgae (Meyer and Griffiths, 2013; Clement et al., 2017; Wei et al., 2019). Henceforth, understanding the photosynthetic carbon partitioning and metabolic regulation in response to very-low CO_2_ (VLC; 300 ppm, or 0.03%) and high CO_2_ (HC; 30,000 ppm, or 3% v/v) in microalgae (del Campo et al., 2014), may provide targets for rational engineering of individual regulatory hubs for enhanced CO_2_ assimilation. Previous studies have reported that low CO_2_ levels have been associated with the air level of CO_2_ (0.03–0.05% CO_2_); however, CO_2_ levels below 0.02% (generally considered as very low CO_2_) have also been considered as low CO_2_. Moreover, the photosynthetic CO_2_ consumption and assimilation by cells supplemented with atmospheric CO_2_, decrease the actual CO_2_ levels even below the generally considered substantial CO_2_ concentration, and often into the range of very low CO_2_ (Vance and Spalding, 2005; Wang et al., 2015). Hence, understanding the relationship between dissolved inorganic carbon (DIC) (Long et al., 2016) concentration and microalgae growth is also important. As a result, a comprehensive correlation between biomass and DIC should also be studied to enhance the rate of CO_2_ biofixation by microalgae since DIC is the only carbon source which is produced by means of CO_2_ aeration and carbon is the major element that makes up the microalgal biomass (Smith et al., 2015; Chang et al., 2016).

*Microchloropsis gaditana*, previously known as *Nannochloropsis gaditana*, has great potential as industrial strain due to its higher lipid content (Poliner et al., 2018; Schädler et al., 2019) (>50% dcw) and are also capable of converting atmospheric CO_2_ into biomass (Carlozzi, 2003), biofuels (Mujtaba et al., 2012), and biorenewables precursors (Fawley et al., 2015; Lee and Sun, 2019). Studies have shown that increase in biomass productivities can be achieved by enhancing photosynthetic efficiency through supplementation of CO_2_ in *Nannochloropsis* sp. (Jiang et al., 2011). Only recently, efforts to engineer this species have taken place and despite these studies, the global picture of carbon fixation and partitioning (both processes being independent of the other) in this species is not well elucidated. Interestingly, this species lacks pyrenoid wherein the Rubisco is localized, eventually fixing CO_2_ in the organic form. Due to the absence of pyrenoid, the efficiency of carbon assimilation is reduced (Mackinder et al., 2017). In this context, CCM machineries are poorly understood among these microalgae and there is a need to reveal the route of carbon flux and carbon partitioning once these CCMs are active. To illustrate the mechanism, we selected a marine microalga *M. gaditana* NIES 2587, and tracked the metabolomic profiles following a time course pattern of 0, 3, 6, and 9 days to adapt the strain from VLC to HC, paving the way for understanding their metabolic regulatory network for enhanced CO_2_ assimilation and biomass production. These insights on the substrate uptake mechanism in *M.* *gaditana* showed that enhancing photosynthetic efficiency will perhaps lead to increased productivities of biomass, biofuels and biorenewables (B3) in these photosynthetic cell factories.

## Materials and Methods

### Microalgae and Culture Conditions

Marine microalgae *Microchloropsis gaditana* NIES 2587 is procured from Microbial Culture Collection, National Institute for Environmental Studies (NIES), Tsukuba, Japan. The strain was grown in minimal medium F/2 (Guillard and Ryther, 1962) under a light regime of 16:8 h and an illumination of 150 µmol m^−2^ s^−1^ photosynthetically active radiation (PAR) in a multi-cultivator MC 1000-OD (Photon Systems Instruments, Czech Republic) with a flow rate of 800 ml min^−1^ with continuous bubbling of air at 24°C. The composition of F/2 medium components (g L^−1^) is as follows: NaNO_3_-0.075; NaH_2_PO_4_·2H_2_O-0.005; Na_2_SiO_3_·9H_2_O-0.03 in artificial sea water (ASW) prepared using NaCl-24; MgCl_2_·6H_2_O-11; Na_2_SO_4_-4; CaCl_2_·6H_2_O-2; KBr-0.1; H_3_BO_3_-0.03; Na_2_SiO_3_·9H_2_O-0.005; SrCl_2_·6H_2_0-0.04; NaF-0.003; NH_4_NO_3_-0.002; Fe_3_PO_4_·4H_2_O-0.001; trace metals solution (in g L^−1^): 1 ml L^−1^ (ZnSO_4_·7H_2_O-0.023; MnSO_4_.H_2_O-0.152; Na_2_MoO_4_·2H_2_O-0.007; CoSO_4_·7H_2_O-0.014; CuCl_2_·2H_2_O-0.007; Fe(NH_4_)_2_(SO_4_)_2_·6H_2_O-4.6; Na_2_EDTA·2H_2_O-4.4); and vitamin B12*-0.135 mg L^−1^; biotin vitamin solution*-0.025 mg L^−1^; thiamine vitamin solution*-0.335 mg L^−1^ (*added after autoclaving the media) (Shaikh et al., 2019). Methods for cell growth profile and their biomass content were estimated by Guillard and Sieracki (2005) and dry weight (dcw) analysis (Sluiter et al., 2008). Growth rates were obtained using the following equation (Levasseur et al., 1993):

$$K=\frac{\ln\frac{N2}{N1}}{t2-t1}$$

Where, N1 and N2 represent cell counts at initial time (t1) and final time (t2), respectively. Doubling time was calculated depending on the specific growth rate (Duong et al., 2015).

$$Doubling\;time=\frac{\ln2}{K}$$

Cells were grown to mid of the logarithmic phase under photoautotrophic condition in F/2 medium. These samples were centrifuged at 5,000 x*g* and resuspended at a density of 2 × 10^6^ cells ml^−1^ in regular F/2 medium with supplementation of CO_2_ (0.03% CO_2_; VLC) and (3% CO_2_; HC) at the intervals of 0, 3, 6, and 9 days for further qualitative metabolomic analysis and profiling.

### Biochemical Analysis

Biochemical analysis of all the samples were done for analyzing the changes in composition, i.e., total pigments (chlorophyll and carotenoids), proteins, carbohydrates, and lipids subjected to VLC and HC. For estimation of total pigments including chlorophyll and carotenoids, 1 ml of cells were pelleted, followed by resuspension of the culture in absolute methanol (1 ml). The solution was briefly vortexed and incubated at 55°C for 1 h for extraction of all the pigments. Finally, the debris was centrifuged at 5,000 x*g* to separate both the pellet and supernatant. The suspension was measured at optical density (OD) of 470, 652, and 665 nm to calculate total chlorophyll and carotenoid content (Lichtenthaler and Wellburn, 1983).

Estimation of proteins were performed by biuret method with slight modifications. The total soluble proteins in extraction buffer containing 1 N NaOH in 25% methanol were extracted as described in Shaikh et al. (2019). To 2 ml of pelleted culture, 1 ml of extraction buffer was added followed by incubation at 80°C for 15 min. Later, these samples were cooled to room temperature (RT) and the cell debris was removed by centrifugation. One hundred microliter of extract was mixed with 50 µl of CuSO_4_ solution (0.21% CuSO_4_ in 30 g of NaOH in 100-ml water) and left for 10 min at RT, followed by measuring absorbance at 310 nm (Chen and Vaidyanathan, 2013).

Estimation of total carbohydrates was done using modified anthrone method (Schneegurt et al., 1994). Cells (approximately 2 ml) were harvested from cultures by centrifugation, and resuspended in 200 µl of water. Hydrolysis was performed for 1 h in 400 µl of KOH [40% (w/v)] at 90°C. The solutions were cooled followed by addition of two volumes of absolute ethanol for precipitating carbohydrates and left for overnight incubation at −20°C. The precipitate was centrifuged for 30 min and dissolved in concentrated H_2_SO_4_ (100 µl), kept at RT for 10 min, followed by dilution in 900 µl of deionized water. Modified protocol was employed in the assay by including sample blank (containing sample + ethanol + sulfuric acid) for each sample in order to reduce the interference occurring due to solubility of proteins and pigments in alcohol, henceforth reducing the over estimation of carbohydrate content within the samples (Ashwell, 1957). Carbohydrates were measured using glucose as standard by addition of the anthrone reagent (containing 2.0 g L^−1^ of anthrone in concentrated H_2_SO_4_). To these precipitated aliquots (500 µl), 1 ml of anthrone reagent was added and absorbance was measured at 575 nm.

Total lipids was estimated using sulpho-phosphovanillin (SPV) method (Mishra et al., 2014). Phosphovanillin reagent was prepared by initially dissolving 0.6 g of vanillin in 10 ml absolute ethanol. To the same, 90 ml of deionized water was added and stirred continuously. Subsequently 400 ml of concentrated phosphoric acid was added to the mixture, and the resulting reagent was stored in the dark until further use. Briefly, 2 ml of culture was centrifuged and resuspended in 100 µl of deionized water. Later, 2 ml of concentrated H_2_SO_4_ was added and the reaction mixture was heated at 100°C for 10 min. The samples were cooled by incubating in ice for 5 min. Finally, 5 ml of freshly prepared SPV reagent was added to the samples and incubated at 37°C with shaking of 200 rpm for 15 min. Absorbance were read at 530 nm for the lipid quantification of the samples.

### Chlorophyll “a” Fluorescence Measurement

Chlorophyll “a” fluorescence signals were documented using the Dual PAM-100 fluorometer (Heinz Walz, GmbH). For complete oxidation of all the reaction centers samples were incubated in dark for 30 min. A saturation light pulse (6,000 μmol photons m^−2^ s^−1^; λ = 660 nm) was used to determine the *Fm* value, followed by calculation of the maximum photochemical efficiency of PSII (*Fv*/*Fm* = (*Fm-Fo*)/*Fm*) (Zhou et al., 2015; Zhao et al., 2017; Agarwal et al., 2019a). The measurements for fluorescence values were obtained from the experimental datasets for the calculation of quantum yields of photochemical quenching, Y (II); non-photochemical quenching, Y (NPQ); and the energy dissipated as heat or fluorescence, Y (NO), followed by calculation of the PSII operating efficiency by *Fq’*/*Fm’* (Baker, 2008). The experiments were conducted for samples aliquoted on different time intervals, i.e., 0, 3, 6, and 9 days with three biological replicates (n = 3) and a minimum chlorophyll concentration of 40 μg ml^−1^ for all the measurements.

### Lipid Quantification and Profiling

For fatty acid methyl esters (FAMEs) analysis in *M. gaditana*, approximately 1 × 10^8^ total cells were hydrolyzed and methyl-esterified in 300 μl of 2% H_2_SO_4_ in methanol for 2 h at 80°C. Prior to the reaction, 50 μg of heptadecanoic acid (Alfa Aesar, USA) was added as internal standard. After esterification step, 300 μl of 0.9% (w/v) NaCl solution and 300 μl of hexane were added and mixed thoroughly for 20 s. To separate both the phases, samples were centrifuged at 3,000 x*g* for 3 min. One microlitre of hexane layer was injected into a 7890A gas chromatography (Kaczur et al.) - mass spectrometry (MS) system (Agilent 7000 GC/MS triple quadrupole system) (Schuhmann et al., 2014; Duong et al., 2015). The running conditions for GC-MS were described by Agilent’s RTL DBWax method (Brown, 1991; Shaikh et al., 2019).

### Qualitative Metabolomics

For extraction of cellular metabolites, approximately, 1 × 10^9^ cells were centrifuged at 8000 ×*g* for 10 min at 4°C and immediately quenched in liquid nitrogen. Further, 1 ml of ice-cold methanol/ethanol/chloroform (2:6:2) was added to the cells for resuspension, followed by sonication of resuspended cells in sonication bath for 15 min (Hirth et al., 2017). Later, these samples were centrifuged at 10,000 ×*g* for 15 min at 4°C and the filtration of supernatant was done by 0.2 µm filter. From the above supernatant (100 µl) was taken and dried by purging nitrogen gas. The dried leftover was dissolved in 0.01 ml of methoxyamine hydrochloride solution [4% (w/v) in pyridine], followed by incubation for 90 min at 30°C by shaking. To the above solution, 0.09 ml of N-methyl-N-(trimethylsilyl) trifluoroacetamide was added and incubated at 37°C for 30 min. The samples were centrifuged at 14,000 ×*g* for 3 min, and the supernatant was taken for the GC-MS/MS analysis (Shaikh et al., 2019). These derivatized metabolite samples were injected in split mode (1:5) into a Agilent 7890A GC-MS equipped with Agilent DB-5 (30 m × 0.25 mm × 0.25 μm) column with the injection port temperature set at 250°C. The GC was operated at constant flow of 1 ml/min helium. The temperature program was started at 60°C for 3 min isothermal and ramping at 5°C/min to 180°C, 3 min isothermal and finally ramping at 10°C/min to 310°C. Data acquisition was performed on a Agilent 7000D Triple Quadrupole mass selective detector with a scan range from 50 to 600 amu. For identification and alignment, peaks were matched against NIST library based on their retention indices and mass spectral similarities (those hits having R value >600 were selected). All the samples were normalized by cell number (10^9^ cells/ml) and the final analysis was done using MetaboAnalyst 4.0 (http://www.metaboanalyst.ca) (Chong et al., 2018).

### Statistical Analysis

The experiments were carried out in biological triplicates, and their standard errors (SEs) were calculated representing mean of three values each. All the data has been represented in terms of mean ± SE (representing “SE” as the standard error for experiments) and plotted graphs by MS Excel software (Microsoft Corporation, USA).

## Results

### Growth Profile and Biomass Yields Subjected to VLC and HC in *M. gaditana*

**Figure 1:**
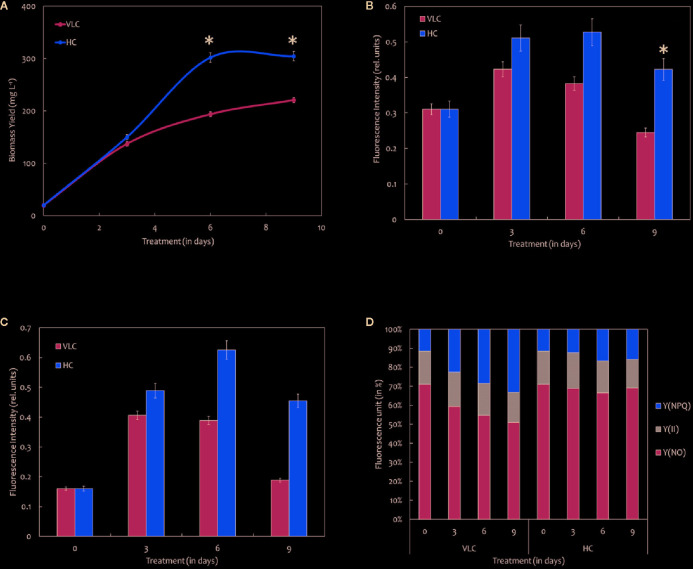
**(A)** Biomass yields of *M. gaditana* at 150 μE of light intensity subjected to VLC and HC supplementation. **(B)** Bar diagram indicating maximum quantum efficiency of PSII photochemistry, i.e., the Fv/Fm ratio in VLC and HC. It is the maximum efficiency at which light absorbed by PSII is used for reduction of Q_A_ (Primary acceptor plastoquinones). **(C)** Bar diagram indicating the PSII operating efficiency,.i.e., the Fq'/Fm' ratio. It estimates the efficiency at which light absorbed by PS II is used for Q_A_ reduction. **(D)** Quantum yields of photochemical quenching Y (II), non-photochemical quenching Y (NPQ) and the non-light induced energy dissipated as heat or fluorescence Y (NO). * indicate statistical significance by one-way ANOVA, p-value < 0.05.

The microalgal strains *M. gaditana* NIES 2587 was cultivated with an initial cell concentration of 5 × 10^6^ cells ml^−1^ in F/2 medium, respectively, in the presence of VLC (0.03%) and HC (3%) till mid- to late-logarithmic phase. In VLC, *M. gaditana* reached a biomass yield of 0.22 g L^−1^ at the end of the 9^th^ day with a specific growth rate of 0.6 day^−1^ (started with an initial inoculum of 0.02 g L^−1^), whereas in HC the biomass yields increased up to 1.5 fold and reached to biomass of 0.3 g L^−1^ within 6 days (**Figure 1A**), thus reducing the overall doubling time by nearly 3 h, i.e., 27.4 to 24.2 h (**Table 1**). Our data presumes that during HC, the *M. gaditana* showed an increase in biomass till 6^th^ day, later slowed down due to photo-saturation and may be the inability to assimilate CO_2_ continuously after 6^th^ day. Henceforth, these clues may provide facts for further investigation of mechanism involved in photosynthetic carbon partitioning due to varying CO_2_ concentrations.

**Table 1:** The different growth parameters of *M. gaditana*.

Treatment(CO_2_)	Specific Growth Rate(d^−1^)	Doubling Time(h)	Biomass Productivity(mg L^−1^d^−1^)
VLC	0.61 ± 0.03	27.42 ± 0.2	30.10 ± 0.25
HC	0.69 ± 0.02	24.27 ± 0.1	47.25 ± 0.18

### Changes Involved in Chlorophyll “a” Fluorescence

Chlorophyll a fluorescence is a fast, non-invasive, precise, and definitive method that remains to be used in a significant number of studies related to photosynthesis (Zhou et al., 2015). In the present study, we have measured the Chl “a” fluorescence in *M. gaditana* grown under VLC and HC conditions. Subsequently, the data analysis and interpretation allow us to characterize the PSII activity and shows strong similarities as indicated in their growth patterns. The maximum quantum efficiency of PSII photochemistry (*Fv/Fm*) of the cultures grown in VLC are compared to HC (**Figure 1B**). The data predicts that the photosynthetic machinery of *M. gaditana* in response to HC is highly active rather than VLC, which demonstrates lowering of doubling time and enhanced growth rates. However, the *Fv/Fm* ratio declines after the 6^th^ day in *M. gaditana*, demonstrating sudden drop in the photosynthetic quantum efficiency (Emerson and Arnold, 1932) of the cells, which has also been reflected as saturation in the growth pattern (**Figures 1A, B**).

**Figure 1C** represents the PSII operating efficiency, that provides an essential approximation of the quantum yield of linear electron flux through the PSII. The PSII operating efficiency is higher in HC than in VLC, and also corresponds with the photosynthetic quantum efficiency of the PSII photochemistry which is higher in HC and is analogous to the growth curve.

There have been numerous reports on the quantum yields of photochemical Y (II) and non-photochemical [Y(NPQ)] quenching; however, there is a limitation on the information available on the Y(NO) significance. It is the energy that is dissipated passively, when the PSII reaction centers are closed (Schreiber and Klughammer, 2008). **Figure 1D** demonstrates a stacked bar graph corresponding to the Y (II), Y(NPQ), and Y(NO). The quantum yield of Y (II) of both VLC and HC cells remain similar throughout the time-course, however, the Y(NPQ) keep on increasing for cells growing in VLC condition.

### Biochemical Analysis

**Figure 2:**
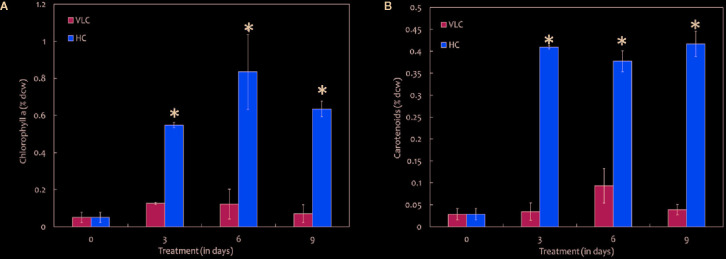
**(A)** Bar diagram representing the time-course dynamics of chlorophyll a content (in % dcw) in *M. gaditana* supplemented with VLC and HC. **(B)** Time-course variation in the carotenoid content (in % dcw) of *M. gaditana* supplemented with VLC and HC. * indicate statistical significance by one-way ANOVA, p-value < 0.05.

To understand the effect of CO_2_ on molecular profiling in *M. gaditana* different biochemical constituents were investigated. **Table 2** shows the biochemical composition, i.e., total pigments (chlorophyll and carotenoids), proteins, carbohydrates and lipids (% dcw) in *M. gaditana*, subjected to VLC and HC conditions. The chlorophyll and carotenoid contents remain to be higher in HC than VLC. It has been previously reported that CO_2_ supplementation increases the growth and chlorophyll contents within the photosynthetic organisms (Husemann and Barz, 1977; Chinnasamy et al., 2009; Wong et al., 2013). In this study, **Figure 2A** shows that the chlorophyll content increases almost by four-fold whereas carotenoid content (**Figure 2B**) enhances by eight-fold within the cells in HC as compared to VLC.

**Table 2:** The biochemical profile of *M. gaditana* on 9^th^ day at 150 μE of light intensity subjected to 0.03% (VLC) and 3% (HC) supplementation.

Strain		Proteins	Lipids	Carbohydrates	Pigments
		(% dcw)			
*M. gaditana*	VLC	40.0 ± 1.2	30.6 ± 0.9	17.7 ± 1.3	0.1 ± 0.0
	HC	41.4 ± 1.0	38.5 ± 1.1	13.5 ± 1.5	1.0 ± 0.0

The total protein content has no drastic effect in *M. gaditana* in both VLC and HC conditions, i.e., nearly consists of 41.4 (% dcw). Moreover, the total lipids content have increased from 30.6 (% dcw) in VLC to 38.5 (% dcw) in HC, whereas the total carbohydrates were declined from 17.7 (% dcw) in VLC to 13.54 (% dcw) in HC; suggesting the diversion of the carbon flux toward lipid (FAMEs) accumulation in *M. gaditana* when subjected to higher CO_2_ conditions.

### Lipid (FAMEs) Analysis and Profiling

**Figure 3:**
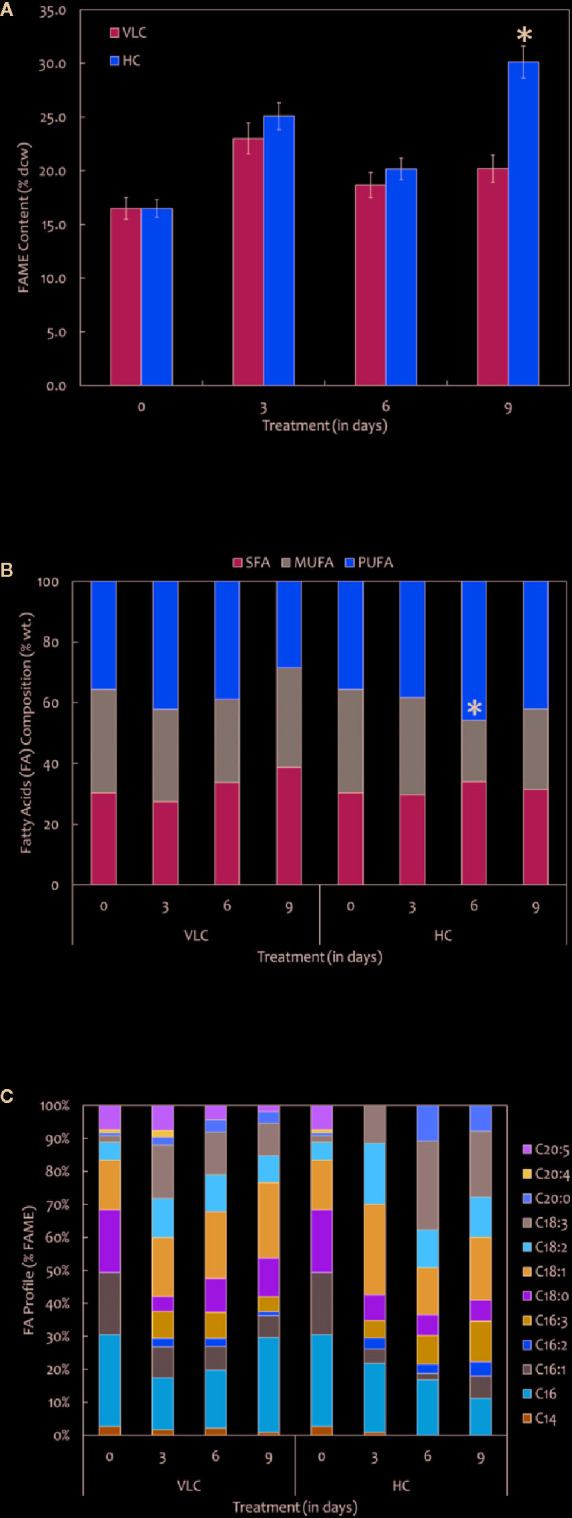
**(A)** Quantitative analysis of total fatty acid methyl esters (FAMEs) in *M. gaditana* (in % dcw) supplemented with VLC and HC. **(B)** Bar diagram representing the saturation and unsaturation ratio of fatty acids (% of FAME) in *M. gaditana* (SFA, saturated fatty acids; MUFA, mono-unsaturated fatty acids; PUFA, poly-unsaturated fatty acids). **(C)** FAME profile depicting the distribution of individual fatty acids in *M. gaditana* subjected to VLC and HC. * indicate statistical significance by one-way ANOVA, p-value < 0.05.

FAMEs were extracted according to the modified Bligh and Dyer procedure and was quantified using GC-MS/MS as described in *Materials and Methods*. The FAME content and profiles varied in response to VLC and HC, respectively. Our data demonstrates overall increase in the total FAME content (% dcw) reaching up to 30% of dcw on 9^th^ day in HC condition (**Figure 3A**). **Figure 3A** represents total FAME content in % dcw, while **Figure 3B** is a representation of FA composition in the form of % wt. of total lipids. The total FAME content remained stable till 6^th^ day while it showed increasing trend at day 9 in high CO_2_ condition (**Figure 3A**). Our results demonstrate reshuffling/shift in fatty acids profiles especially in PUFAs (% wt of total lipids) within the cells on day 6 in HC (**Figure 3B**). However, overall increase in the total FAME content was observed on day 9 in HC condition while no change was observed in saturation and unsaturation profiles. The time-course also demonstrates that the PUFA content in VLC conditions were decreased substantially from 3^rd^ day to 9^th^ day (42.1% dcw to 28.3% dcw), diverting the carbon flux toward the accumulation of monounsaturated fatty acids (MUFAs), whereas in HC, the PUFA content increases drastically from 35% dcw to 45% dcw by 6^th^ day. Also during the HC conditions, the FAME profile (**Figure 3C**) demonstrates that the relative content of C18:2 (linoleic acid) and C18:3 (linolenic acid) is higher in *M. gaditana*; however, C18:1 (oleic acid) and C16:0 (palmitic acid) are the major FAMEs present in VLC conditions, implying that the activity of desaturases involved in lipid metabolism may be upregulated at higher CO_2_ concentrations.

### Metabolome Analyses

The CCMs in microalgae are essential for the photosynthetic processes and survival at low CO_2_ environments (Wang et al., 2015). CCMs in photosynthetic organisms operate to enable the assimilation of CO_2_ with the help of active inorganic carbon (Ci) uptake systems and increased carbonic anhydrase activity to raise Ci accumulation within the cells when inorganic carbon (Ci) is limiting through the dehydration of accumulated bicarbonate (Spalding, 2008; Beardall and Raven, 2016). There are a number of reports in the model microalga, *C. reinhardtii* about the transporters involved in the transfer of the inorganic carbon pool toward Rubisco, however, not much is known about the dynamics of the metabolites inside the cell and the specific knowledge regarding the metabolomic profiles remains elusive (Duanmu et al., 2009; Yamano et al., 2015; Shaikh et al., 2019). In this study, we have employed qualitative metabolomics in order to understand the changes in the metabolomic profiles inside the cell when subjected to VLC and HC supplementation, which will provide new insights and understanding regarding the photosynthetic carbon partitioning and metabolic regulation in *M. gaditana*. The metabolite extraction and derivatization of *M. gaditana* was carried as described earlier in *Materials and Methods*. The major advantage with a metabolomics approach is the non-biased information about pool sizes in a large number of metabolites under particular conditions (Renberg et al., 2010). Metabolomics datasets were analyzed for the time-course experiments, resulting in nearly a total of 40 GC-MS peaks. As a result of alternate derivatization, repetition of identical metabolites was observed to be very common in the raw data files; such metabolites were eliminated if not significant, and the peaks were manually curated to obtain 31 metabolites that were further processed (Hirth et al., 2017; Shaikh et al., 2019).

**Figure 4:**
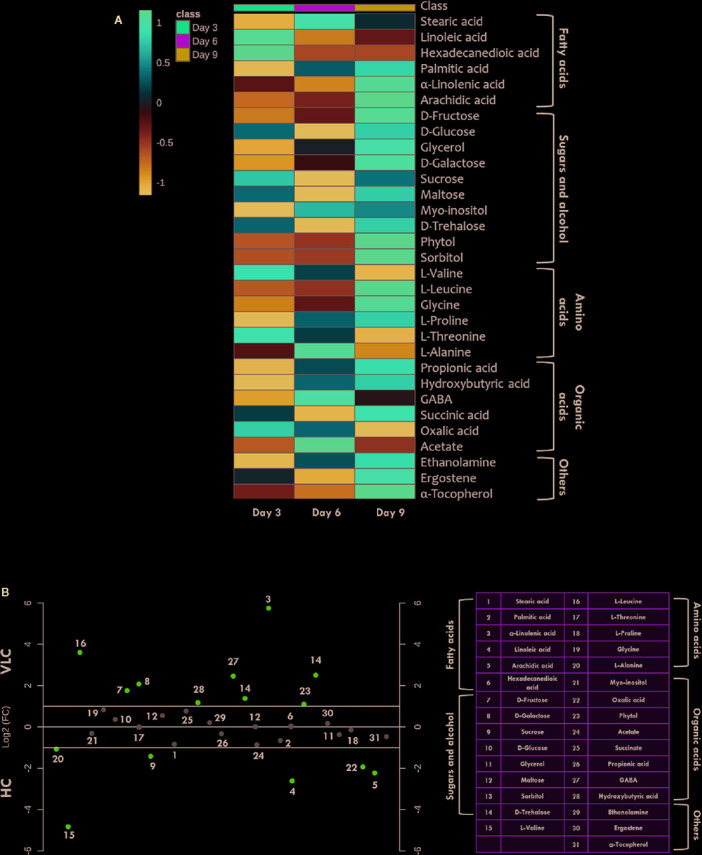
Time-course metabolome dynamics in *M. gaditana* under VLC/HC. **(A)** Heatmap represents the log_2_ fold change of all metabolites (represents time-course, i.e., 3, 6, and 9 days) in VLC/HC. **(B)** Dot-plot representing the fold-change of different metabolites in VLC and HC supplementation on 9^th^ day in *M. gaditana* (pink dots represent the metabolites which show fold change >2; gray dots represent the metabolites fold change < 2).

**Figure 4A** represents heatmap showing time-course log_2_ fold changes of metabolites in VLC/HC conditions that were either upregulated or downregulated, wherein the legend scale denotes as follows: red color for upregulation while blue indicates downregulation. It is evident that several metabolites show a dynamic alteration in both VLC and HC conditions. Relative abundances of nearly 31 metabolites were obtained, and most of them belong to either sugars, or/and fatty acids. In *M. gaditana*, metabolites such as fructose, glucose, galactose, and phytol were increased in VLC conditions, whereas sucrose is significantly upregulated in HC supplementation. It is also evident from the dot-plot (**Figure 4B**) that metabolites such as sugars and alcohols namely fructose, glucose, phytol, trehalose, and galactose were upregulated on the 9^th^ day when subjected to VLC. Furthermore, fatty acids such as linoleic acid and arachidic acid were enhanced in HC, with both the metabolites in their higher abundance of more than two-fold change (**Figure 4B**).

Increased levels of these compounds indicate breakdown of starch and the regulation of maltose metabolism, respectively. This physiological behavior of cells is reported to be an adaptive strategy as a result of environmental stress (Agarwal et al., 2019b). On the other hand, TCA cycle intermediate such as acetate seems to be upregulated on 3^rd^ day as compared to days 6 and 9 (**Figure 4A**) when subjected to HC. Fatty acids such as stearic acid, hexadecanedioic acid palmitic acid, linoleic acid and arachidic acid were enhanced in HC indicating the diversion of carbon flux from TCA cycle to lipid biosynthesis. As depicted in **Figure 4B**, another metabolite which is upregulated in HC is α-tocopherol, is a secondary metabolite. The primary role of α-tocopherol is scavenging of reactive oxygen species (ROS) within the cells. Certain amino acids such as L-valine, L-alanine, and L-proline were also increased (**Figures 4A, B**), indicating the diversion of flux through intermediates of the TCA cycle, ultimately leading toward protein synthesis. Our data demonstrates significant molecular alteration in the relative abundance of various metabolites in response to VLC and HC, allowing us to predict a metabolic route in the diversion of the carbon flux in *M. gaditana* when subjected to VLC and HC conditions.

## Discussion and Conclusions

Aqueous environments are directly not favorable conditions for efficient photosynthesis due to the CO_2_ diffusion rate, i.e., 10,000-fold lower than in atmospheric conditions (Yamano et al., 2015). However, the aquatic photosynthesis of microalgae accounts for a large proportion (nearly 50%) of the global primary productivity (Giordano et al., 2005). The cellular physiology governs the channelization of the carbon flux toward biomass and biosynthesis of energy storage molecules. Depending on the requirement and the environmental perturbations, there is a metabolic shift that can alter the route of photosynthetically assimilated inorganic carbon from the synthesis of biomass to production of storage molecules, such as lipids and other high-value biorenewables (Valenzuela et al., 2012; Fields et al., 2014; Schuhmann et al., 2014; Shaikh et al., 2019). Major algal research has been primarily focused on lipid production subjected to nitrogen stress while compromising biomass productivities (Alboresi et al., 2016; Shaikh et al., 2019). Improving the biomass productivity of microalgal strains is a major factor that would facilitate the economic viability of algal biofuels (Davis et al., 2011; Hildebrand et al., 2013). It has been shown that light can induce lipid accumulation and potentially is the master regulator of metabolism; however, it is important to understand how different organisms regulate the carbon flux toward lipid or/and carbohydrate biosynthesis (Sforza et al., 2012; Alboresi et al., 2016). Microalgae have adapted different strategies to cope with the environmental stress and divert the carbon flux with the help of a photosynthetic carbon assimilation process, i.e., CCM (Wang et al., 2015). The CCMs are the fundamental process in algal photosynthesis, metabolism, growth, and biomass production and much of the work related to CCMs has been reported in the model microalga, *C. reinhardtii*. Unlike the CO_2_ enrichment in C4 plants, CCMs in these aquatic microalgae function intracellularly by generating a reservoir of DIC, the uptake of this pool of DIC is propelled by energy-coupled Ci transport systems (Wang and Spalding, 2006). In VLC conditions, cells undergo dynamic ultrastructural changes as well as induce additional CCM-based proteins. This leads to the acclimation of the photosynthetic machinery by regulating the ATP supply to assist the transfer of Ci into the cells eventually enhancing the process of photosynthesis (Ramazanov et al., 1994; Geraghty and Spalding, 1996; Renberg et al., 2010). In *C. reinhardtii* grown under CO_2_-limiting conditions, the major pool of Rubisco is localized in a central structure inside the chloroplast known as?A3B2 show [#,32] ?> pyrenoid (Badger et al., 1998). Although there have been advances about the understanding of the CCMs in *C. reinhardtii* and diatoms, extensive experimental studies have been limited to only these selected taxa. The marine microalga, *M. gaditana*, has emerged as an industrially relevant organism due to their oleaginous nature (Hu et al., 2008; Varfolomeev and Wasserman, 2011; Ratha and Prasanna, 2012). However, there is very little information available about the CCMs of *M. gaditana*, more importantly, they appear to lack pyrenoids, and the role of several metabolite pools inside the cell governing the diversion of carbon flux (Gee and Niyogi, 2017) seems to be elusive. Hence, to solve such challenge, insights into the CO_2_ uptake and assimilatory machineries with regards to the CCMs are essential for understanding the physiological and metabolic patterns of these cell factories.

In the present work, we have demonstrated the growth and cellular physiology to improve our understanding regarding the CCM of *M. gaditana* in response to VLC and HC. Biomass concentration increases under VLC and reaches the maximum level on the last day of cultivation, i.e., 9^th^ day. On the other hand, the growth saturates in *M. gaditana* when subjected to HC after the 6^th^ day. The biomass concentration, although, under HC is always higher than under VLC, which is also consistent with previous reports showing an increased biomass production at higher CO_2_ concentrations (Sforza et al., 2012; Adamczyk et al., 2016). It has been reported that CO_2_ biofixation can be increased by using a higher concentration of biomass with the same volume of culture (Adamczyk et al., 2016). VLC and HC have a significant effect on the photochemical efficiencies in *M. gaditana*, as demonstrated by the Fv/Fm ratios. The maximum photochemical efficiency of PSII (Fv/Fm) under HC is higher than in VLC. A higher Fv/Fm ratio is usually interpreted as higher photosynthetic performance while a lower ratio represents the photoinhibition of PSII. It has been reported that the higher Fv/Fm ratio is also due to the influence of certain pigments on the photosystems (Zhou et al., 2015; Zhao et al., 2017; Agarwal et al., 2019a). Our results suggest that the cells cultivated in HC undergo more physiological changes than in VLC conditions. The decline of photosynthetic activity may be due to decrease in chlorophyll contents in both VLC and HC after 6^th^ day. The decrease in Fv/Fm ratio is often interpreted as photo-damage which might be caused because of the inactivation of PSII reaction centers. This is caused due to an inhibition of electron transport at both donor and acceptor sites of PSII further promoting a dissemination of excitation energy in favor of PSI, resulting in an increase in the electron flow near PSI (Lu and Vonshak, 2002; Rym, 2012). This decline represents a defensive process with the dissipation of excess energy from the photosystems which is usually identified as an adaptive acclimation system to down regulate PSII. The alterations in PSII photochemical reactions suggests that there is a decline in the ability of the photosynthetic apparatus to maintain the oxidative state of Q_A_ and is highly prominent in VLC starting from day 6.

It is evident (**Figure 1D**) that there is a constant decline in Y(NO) till the 9^th^ day in VLC cells. Y(NO) is generally considered as a straightforward indicator of the reduction state of plastoquinones present in the membranes (Grieco et al., 2012). Consequently, it is assumed that the HC cells retained the ability to efficiently regulate the photosynthetic electron transport chain. During stress phenomenon, the increase in non-photochemical quenching can often be accompanied by photo-inactivation of PSII reaction centers, which will dissipate excitation energy as heat (Maxwell and Johnson, 2000). Photo-inactivation can lead to oxidative damage and loss of PSII reaction centers (Baker, 2008), both of which are associated with decrease in Fv/Fm ratios. Microalgae tend to dissipate excess energy in the form of heat (Agarwal et al., 2019a). Similar phenomenon in dissipation of the activation energy as Y(NPQ) in VLC cells might be a survival mechanism that result in the reduction of overall cell biomass.

Biochemical changes in response to VLC and HC conditions where the major shift has been observed in production of lipids and total pigments. To utilize the carbon efficiently, algal cells must maintain adequate chlorophyll and carotenoid levels (**Figures 2A, B**). Increased levels of phytol in VLC indicate breakdown of chlorophyll (Wase et al., 2017), which can be seen in **Figure 2A** and hence the reduced levels of photosynthetic efficiency (**Figure 1B**). The main component affected by CO_2_ supplementation is the photosynthetic machinery and the increase in total chlorophyll and carotenoid content reflects the enhanced photosynthetic efficiency in the presence of HC. The total proteins remain almost constant and no drastic changes were observed in HC of *M. gaditana.* The supplementation of CO_2_ has been known to have a profound effect on the two energy rich storage molecules, lipids, and carbohydrates (Wang and Spalding, 2014). The lipid content of the oleaginous microalga *M. gaditana* increased by 1.3-fold in response to HC. Previous studies have demonstrated that excess of carbon will be directed toward lipid synthesis, suggesting the channelization of carbon toward acetyl-CoA in response to elevated concentration of CO_2_ (Valenzuela et al., 2012; Sun et al., 2016). The increased amount of the FAMEs in response to carbon supplementation are higher due to conversion of CO_2_ into acetyl CoA, which provides the precursor molecules for the biosynthesis of lipids. Also, the increased photosynthetic efficiency of *M. gaditana* under HC implies more assimilation of carbon photosynthetically, that could be redirected into neutral lipid biosynthesis (Renberg et al., 2010; Prathima Devi et al., 2012; Schuhmann et al., 2014). Due to oleaginous nature of the strain most of the carbon flux tends to divert toward lipids rather than carbohydrate production. Whereas in low CO_2_ the strains prefer to synthesize low energy molecules, i.e., carbohydrates such as laminarin (Vogler et al., 2018).

The major strategy to increase the amount of non-polar triacylglycerols (TAGs) is limiting the concentration of nitrogen and/or phosphate in the medium; however, a minimal concentration of the carbon should be maintained in the medium, as it is an indispensable substrate for the biosynthesis of relevant biomolecules (Spalding, 2008). Therefore, one of the common solutions that can be implied here will be removal and/or presence of minimal nutrients with increased levels of inorganic carbon in the medium. This will enhance the overall lipid productivities in many microalgal species (Sharma et al., 2012). The combined effect of nutrient deficiency and inorganic carbon supplementation has been employed earlier to enhance the lipid productivity in *C. reinhardtii*, *Scenedesmus obtusiusculus*, and *Chlorocuccum littorale* (Toledo-Cervantes et al., 2013; Fields et al., 2014; Singh and Singh, 2014). Numerous studies have examined lipid accumulation in green algae for cells grown under the presence of a variety of stress conditions such as nitrogen, sulfur, phosphorus, or iron deprivation. Wase et al. employed omics technologies to detect key variations in the metabolic networks and regulatory components that contribute to lipid biosynthesis (Wase et al., 2014; Wase et al., 2019). The total FAME production in *M. gaditana* showed a substantial increase starting from 3^rd^ day onward. However, the overall FAME content may vary when subjected to HC conditions. With supplementation of the inorganic carbon, the FAME profiles in *M. gaditana* increased substantially with the accumulation of up to nearly 30% (dcw) neutral lipids on the 9^th^ day.

Our preliminary data using qualitative metabolomics suggests increase in the acetate levels when subjected to HC condition, eventually leading to the fatty acid biosynthesis. Studies have shown that increase in acetate concentration may lead to elevated activity of pyruvate dehydrogenase (PDH), the enzyme that bridges the glycolysis and lipid biosynthesis pathways within the plastids (Sun et al., 2016). Also, previous studies have demonstrated that the inhibition of PDH blocks lipid production in *C. sorokiniana* whereas silencing of PDH kinase (negative regulator of PDH) in *N. salina* enhances TAG biosynthesis (Sun et al., 2016; Ma et al., 2017). Henceforth, increase in acetate concentration and synergetic activity of PDH may be responsible for the overall increase in the total FAME content, when supplemented with higher CO_2_ (30,000 ppm). The FAME profile depicts a unique pattern in *M. gaditana* showing maximum PUFA content and later rapidly declines in response to very-low level CO_2_ suggesting the inhibition of the desaturase activity in the lipid metabolism. However, when exposed to HC, the increased photosynthetic efficiency resulted in increased pool of NADPH and evolution of O_2_ which is required for the desaturase enzyme in the biosynthesis of polyunsaturated fatty acids, and hence, there is increase in linoleic acid and linolenic acid contents. Overall, the dynamics of the biochemical parameters such as chlorophyll and lipid molecules suggest the diversion of the carbon fixation and assimilation pathways favoring enhanced photosynthetic efficiency.

The schematics of compartmentalization along with gene editing have a huge impact on the metabolic capabilities of microalgal cells in diverting the carbon flux *via* alternative routes (Ginger et al., 2010; Martin, 2010; Hildebrand et al., 2013). Proteomic approaches have been known to be powerful; however, it has been reported that the metabolic flux cannot be associated with the concentration of proteins inside the cell (Villiers et al., 2011). There have been a numerous reports depicting the importance of metabolomic approach to unravel the mechanism of biological processes in response to several factors (such as nitrogen deficiency, iron deficiency, etc.) in plant systems (Rellán-Álvarez et al., 2010; Zhang et al., 2010; Osorio et al., 2011; Weckwerth, 2011; Amiour et al., 2012). To illustrate the different diversion routes of carbon flux in VLC and HC, the relative abundances of the metabolite profiles were analyzed. Overall, a total number of 31 metabolites were obtained in *M. gaditana* when subjected to VLC and HC, with few metabolites that were common in both the conditions. Primary sugars such as glucose, galactose, trehalose, etc., demonstrated highest variation in the metabolomic profiles in VLC. When the metabolome profile of VLC is compared with HC, the accumulation of glucose, trehalose and galactose indicate a constitutive carbohydrate metabolism in VLC conditions. Trehalose, a non-reducing disaccharide is known to be a stabilizing agent for protecting the membranes against impairment, and help retain cellular integrity (Wase et al., 2014; Agarwal et al., 2019b). Increase in the level of maltose was observed which acts as a transitory product of carbohydrate metabolism that is reported to be produced as a result of the starch breakdown machinery (Weise et al., 2005). Increase in maltose levels also indicate the onset of photo-respiratory conditions, which explains the low Fv/Fm ratio of the cells under VLC (Weise et al., 2006). Also, the biochemical profile of *M. gaditana* indicate that the carbohydrate production increases in VLC which is in strong co-relation with the metabolome profile. It has been observed that the amino acids being the primary constituents for the protein synthesis are significantly increasing. But these amino acids have several secondary functions under tightly controlled steady state levels such as functioning as signaling molecules and precursors for the biosynthesis of phytohormones and secondary metabolites (Hildebrandt et al., 2015). As a result, the increase in the amino acids in HC is not reflected in the total protein content. Amino acids such as proline, valine, and alanine show an increase in their accumulation under HC, wherein, proline is seen to be accumulated under stress conditions and is also reported to maintain the osmoregulation inside the cell (Hayat et al., 2012). Also, no change in overall protein content suggests that the normal expression of the native proteins is sufficient to perform the metabolic reactions.

It has also been observed that the accumulation of α-tocopherol increases in cells supplemented with CO_2_ with increased production of lipids during HC condition. The increase in lipid production in HC on the 9^th^ day can also be attributed to the presence of α-tocopherol since it has been reported to be inhibiting lipid degradation. It has been shown that tocopherols protect the dormant and germinating seeds in *A. thaliana* against oxidative degradation of lipids (Sakuragi et al., 2006). The elevated levels of α-tocopherol in the chloroplast membranes can also be associated to the ability of tocopherols to quench ROS, hence protecting the photosynthetic apparatus from oxygen toxicity (Fachechi et al., 2007). It is also reported to be involved in the regulation of photosynthesis and macronutrient uptake and utilization (Sakuragi et al., 2006; Fritsche et al., 2017; Shaikh et al., 2019). The upregulation of propionic acid in HC is in correlation with the presence of valine, which is shown to be involved in the synthesis of succinyl coA, an intermediate in the TCA cycle in photosynthetic organisms (Pan et al., 2017). Consequently, the carbon flux is diverted toward lipid biosynthesis through the TCA cycle. Acetic acid is the precursor of TCA cycle, which further drives the fatty acid biosynthesis. The upregulation of acetic acid during HC along with several fatty acids such as stearic acid, propionic acid, palmitic acid, linoleic and arachidic acids, indicates that HC promotes the growth of the cell as well as the production of energy storage molecules, i.e., lipids.

**Figure 5:**
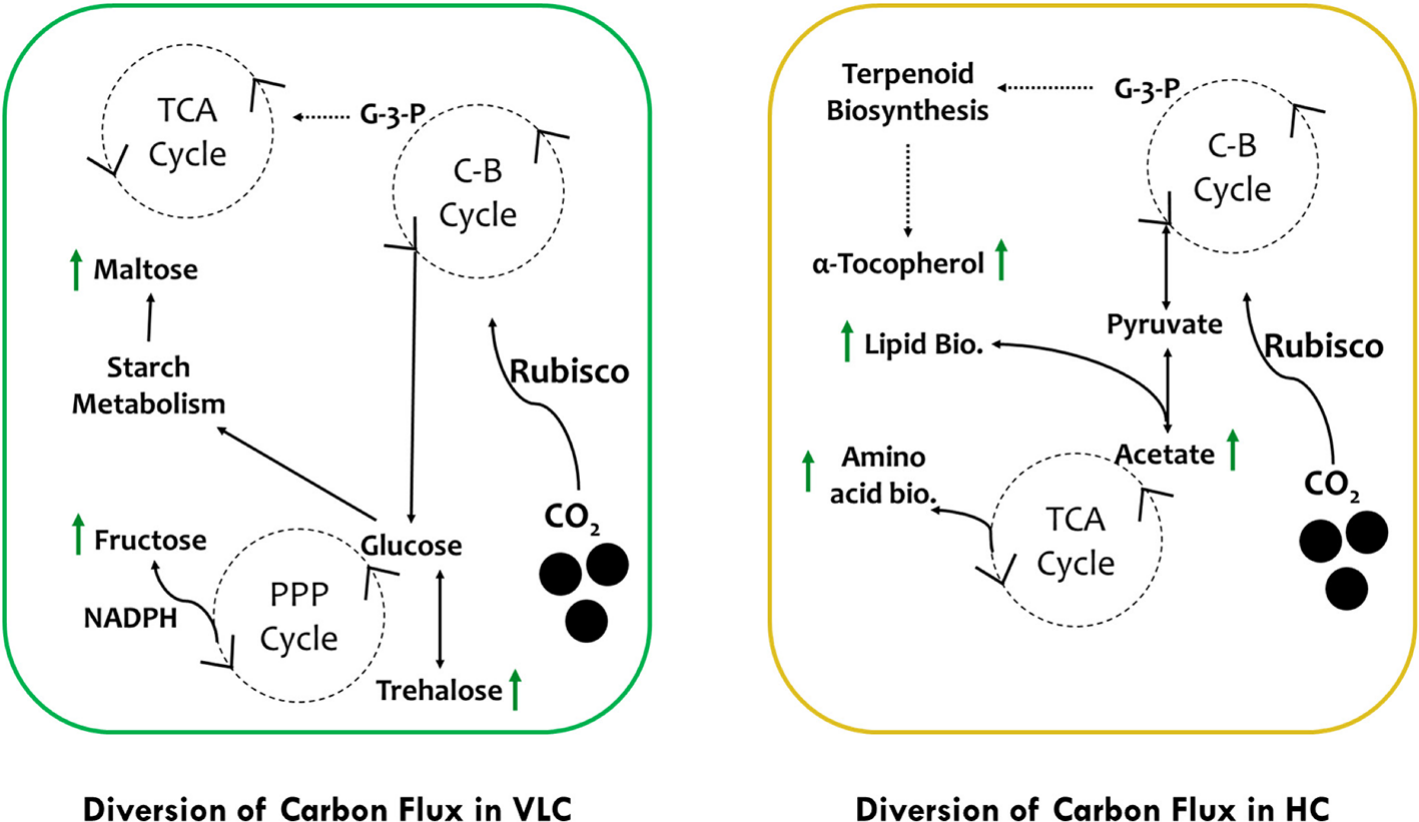
Schematic representation of photosynthetic carbon partitioning and metabolic regulation routes of carbon flux subjected to VLC and HC supplementation in *M. gaditana*.

In conclusion, we would like to hypothesize two separate pathways of carbon flux in VLC and HC (**Figure 5**). In VLC, the carbon flux inside the cells is diverted in such a way that carbohydrate metabolism functions in a constitutive manner, generating the energy currency through the pentose phosphate pathway leading to starch breakdown and formation of maltose. In HC, the TCA cycle is upregulated further diverting the carbon flux toward lipid biosynthesis. Moreover, pyrenoid lacking microalgae such as *Pavlova lutheri* have higher amount of Rubisco (Heureux et al., 2017). In *Microchloropsis gaditana*, which also lacks pyrenoid, this enhanced Rubisco may help in assimilation of more carbon at HC that is partitioned toward growth and lipid biosynthesis as observed in the current study. Overall, our preliminary data analysis on supplementation of CO_2_ provides interesting out comes, i.e., in VLC we presume that CCMs are active and leading to accumulation of carbohydrates or/and lipids without compromising growth whereas in HC, CCMs may be inactive or lower in their activities but overall supplementation of higher CO_2_ will enhance productivities in biomass, biofuels, and biorenewables (B3) in *M. gaditana*. In conclusion, based on these preliminary data we would further try to evaluate the role of CCMs and its relation to Rubisco in *M. gaditana* employing multiomics approach to provide insights on the targets responsible for photosynthetic carbon partitioning and metabolic regulation in these green cell factories.

## Data Availability Statement

This data is available at the NIH Common Fund’s National Metabolomics Data Repository (NMDR) website, the Metabolomics Workbench, https://www.metabolomicsworkbench.org, where it has been assigned Project: PR000893, Study ID: ST001395. The data can be accessed directly *via* its Project DOI: 10.21228/M8869V. This work is supported by Department of Biotechnology (DBT), India grant BT/PB/Center/03/2011.

## Author Contributions

MK, AN, and PJ designed the experiment. MK, IM, and KS executed the experiments. AN and PJ supervised the project. MK, IM, and AN wrote the manuscript with all the input from the authors. All authors contributed to the article and approved the submitted version.

## Funding

The work was supported by the grants from the Department of Biotechnology, Government of India, to PJ (Sanction No. BT/PB/Center/03/2011) and to AN (BioCARe Scheme No. BT/PR18491/BIC/101/759/2016). Senior Research Fellowship to MK, IM, and KS from the Department of Biotechnology and University Grants Commission (UGC), Government of India, is duly acknowledged.

## Conflict of Interest

The authors declare that the research was conducted in the absence of any commercial or financial relationships that could be construed as a potential conflict of interest.
